# A systematic review of contamination (aerosol, splatter and droplet generation) associated with oral surgery and its relevance to COVID-19

**DOI:** 10.1038/s41405-020-00053-2

**Published:** 2020-11-24

**Authors:** Jennifer E. Gallagher, Sukriti K.C., Ilona G. Johnson, Waraf Al-Yaseen, Rhiannon Jones, Scott McGregor, Mark Robertson, Rebecca Harris, Nicola Innes, William G. Wade

**Affiliations:** 1 Faculty of Dentistry, Oral & Craniofacial Sciences, King’s College London, Denmark Hill Campus, Bessemer Road, London, SE5 9RS UK; 2 Faculty of Dentistry, Oral & Craniofacial Surgery, King’s College London, Centre for Host Microbiome Interactions, Denmark Hill Campus, Bessemer Road, London, SE5 9RS UK; 3 Cardiff University School of Dentistry, Dental Public Health, Applied Clinical Research and Public Health, Heath Park, Cardiff, CF14 4XY UK; 4 School of Dentistry, Child Dental and Oral Health, University of Dundee, 2 Park Place, Dundee, DD1 4HR UK; 5 College of Biomedical and Life Sciences, School of Dentistry, Cardiff University, Heath Park, Cardiff, CF14 4XY UK; 6 Library and Learning Centre, University of Dundee, Dundee, DD1 4HR UK; 7 Department of Public Health, Policy & Systems, University of Liverpool, Room 124, 1st Floor, Block B, Waterhouse Building, 1-5 Brownlow Street, Liverpool, L69 3GL UK; 8 King’s College London, London, UK

**Keywords:** Infection control in dentistry, Oral diseases

## Abstract

**Introduction:**

The current COVID-19 pandemic caused by the SARS-CoV-2 virus has impacted the delivery of dental care globally and has led to re-evaluation of infection control standards. However, lack of clarity around what is known and unknown regarding droplet and aerosol generation in dentistry (including oral surgery and extractions), and their relative risk to patients and the dental team, necessitates a review of evidence relating to specific dental procedures. This review is part of a wider body of research exploring the evidence on bioaerosols in dentistry and involves detailed consideration of the risk of contamination in relation to oral surgery.

**Methods:**

A comprehensive search of Medline (OVID), Embase (OVID), Cochrane Central Register of Controlled Trials, Scopus, Web of Science, LILACS and ClinicalTrials.Gov was conducted using key terms and MeSH (Medical Subject Headings) words relating to the review questions. Methodological quality including sensitivity was assessed using a schema developed to measure quality aspects of studies using a traffic light system to allow inter- and intra-study overview and comparison. A narrative synthesis was conducted for assessment of the included studies and for the synthesis of results.

**Results:**

Eleven studies on oral surgery (including extractions) were included in the review. They explored microbiological (bacterial and fungal) and blood (visible and/or imperceptible) contamination at the person level (patients, operators and assistants) and/or at a wider environmental level, using settle plates, chemiluminescence reagents or air samplers; all within 1 m of the surgical site. Studies were of generally low to medium quality and highlighted an overall risk of contaminated aerosol, droplet and splatter generation during oral surgery procedures, most notably during removal of impacted teeth using rotatory handpieces. Risk of contamination and spread was increased by factors, including proximity to the operatory site, longer duration of treatment, higher procedural complexity, non-use of an extraoral evacuator and areas involving more frequent contact during treatment.

**Conclusion:**

A risk of contamination (microbiological, visible and imperceptible blood) to patients, dental team members and the clinical environment is present during oral surgery procedures, including routine extractions. However, the extent of contamination has not been explored fully in relation to time and distance. Variability across studies with regards to the analysis methods used and outcome measures makes it difficult to draw robust conclusions. Further studies with improved methodologies, including higher test sensitivity and consideration of viruses, are required to validate these findings.

## Background

The global COVID-19 pandemic caused by the SARS-CoV-2 virus has required professionals, providers and policymakers to urgently revisit infection control procedures,^1–5^ including personal protective equipment (PPE) for the protection of staff and patients across healthcare in general,^6^ and dentistry in particular.^7–10^ Its impact has resulted in reducing the nature and scope of care to the bare minimum in reaction to the first wave of the pandemic.^11^

SARS-CoV-2 shows evidence of transmission by direct contact, droplets and fomites,^12–14^ with increasing emerging evidence suggesting airborne transmission.^15,16^ These are important concerns in dental clinics and hospitals because it is hard to avoid the generation of large amounts of droplets and aerosol that include the patient’s saliva and even blood during all aspects of the dental practice,^12^ and particularly in oral surgery, where both are implicated. A susceptible individual, staff or patient could inhale droplets and/or aerosols, and become infected.^14,16^ As we struggle to develop a deep understanding of this particular virus, it is important to remember that infectious aerosols can be produced by coughing and sneezing,^16^ and even singing has been associated with the transmission of SARS-CoV-2.^17^ Given the emerging role of aerosols involved in SARS-CoV-2 transmission, we need to give due consideration to their relevance for all aspects of dentistry, which involves close contact with the patient and their upper respiratory tract.

Oral surgery ranges from simple extractions through to surgical removal of teeth, such as impacted third molars, to implant surgery, all of which may be provided in primary or secondary care settings. At the height of the pandemic, simple oral surgery (extractions) remained an essential component of urgent care delivery to address pain and infection, even when routine dental services had been halted.^11,18–20^ While dentists in primary care provide routine oral surgery such as extractions, more complex care is increasingly a specialised function,^21,22^ generally delivered by oral or oral and maxillofacial surgeons depending on the organisation of specialist care within the country. Therefore, the risk of SARS-CoV-2 contamination during oral surgery procedures is especially important for oral surgical services.

Dentistry is practised in a contaminated field and has relied on universal precautions for routine care delivery. Universal precautions have proved sufficient to prevent transmission of infectious disease spread by droplets (particles >5 µm in diameter), as demonstrated by circumstantial evidence and real-life experience. However, in the current pandemic, the importance of understanding the risk of exposure through both droplets and aerosol (particles ≤ 5 µm) has been realised and the historical wealth of evidence for dentistry revisited.^23,24^ For oral surgery, the risks of transmission via blood, following concerns over blood-borne viruses such as HIV and hepatitis B and C, have resulted in clinicians regularly wearing standard surgical PPE in recent decades, rather than merely relying on universal precautions.

It is increasingly recognised that particle size is the most important determinant of aerosol behaviour.^16,17^ Small particles may be immediately inhaled, but biological factors such as the size of the inoculum, survival of desiccation and wider environmental factors, including humidity, temperature and air movement, impact contamination, together with the defences of the host influence their impact.^16^ Pathogens have been identified in aerosols,^16^ and this has implications for SARS-CoV-2. Furthermore, dental procedures may produce dental aerosols, which carry an infectious virus, and there is now evidence that aerosols can stay airborne for up to 3 h and probably longer.^25^ A dental aerosol-generating procedure (AGP) performed on an infected individual could therefore produce a local outbreak.

Since there are no generally accepted terms and definitions in this field of study, with no clear delineations between terms frequently used, the following will be used in this paper:

*Splatter*: Mixture of air, water and/or solid substances >50 μm in diameter that are visible to the naked eye and behave in a ballistic or projectile manner.^26,27^

*Droplets*: Inspirable particles >5 μm in diameter, which can be deposited on upper respiratory tract levels and mucosa.^28,29^

*Aerosol*: An aerosol is defined as a suspension of liquid or solid in the air, and while some researchers describe suspensions with particles of up to 50 µm diameter as aerosols, the accepted use of the term in infectious disease research includes only particles ≤5 µm.^14,28^

*Bioaerosol or infectious aerosols*: Aerosol-comprising particles of biological origin or activity, which may affect living things through infectivity, allergenicity, toxicity, pharmacological or other processes.^30,31^

The research question driving this study is: what is known, and what is not known, about bioaerosols relevant to oral surgery? This involved identifying and cataloguing activities within oral surgery delivered in the dental surgery that generates aerosols, splatter and droplets. This will be determined by the presence of contamination as measured by bacterial or fungal colony-forming units (CFUs) on agar culture plates or other measures, including detection of blood, visual and occult.

The objectives of this review follow the wider review,^23,24^ as follows, to:

- Characterise the pattern of aerosol spread and settle relevant to oral surgery in dental surgery.
- Identify whether there is evidence of an association with exposure, infection and transmission of pathogenic microorganisms.
- List microorganisms that have been studied.
- Record outcomes and outcome measures.
- Identify gaps in the evidence in relation to oral surgery.

This is the first of a series of papers reporting the detailed findings by procedure, as part of a wider body of research conducted to provide a deeper understanding of dentistry in light of the current COVID-19 Pandemic. The overarching paper compares procedures and has proposed a hierarchy of risk.^23^ While the overarching data have been reported separately by our research team,^23^ further reports detailing the context of different dental procedures helps enable research to inform future policy and practice.

## Methods

This research is part of a large systematic review registered under the International Prospective Register of Systematic Reviews ID CRD42020193058.^24^ It involved searching of key databases (Medline (OVID), Embase (OVID), Cochrane Central Register of Controlled Trials, Scopus, Web of Science and LILACS) for studies meeting the inclusion criteria, together with ClinicalTrials.gov for any date up to June 2020. The search strategy comprised keywords and MeSH (Medical Subject Headings). Titles and abstracts were deduplicated and screened using Rayyan.^32^ Full-text publications were sought for all papers eligible for inclusion and were managed using the Endnote referencing software. All screening was conducted independently and in duplicate by two reviewers. The set inclusion and exclusion criteria, listed in Annex 1 of the protocol, comprised diverse methodologies, dental settings (hospital, practice and experimental), dental procedures, consideration of aerosols or droplets.^24^

In total, 723 papers were identified after duplicates were removed. A Preferred Reporting Items for Systematic Reviews and Meta-Analyses (PRISMA) flowchart,^33^ detailing the number of studies through each stage of the larger review process, is available as a pre-print online.^23^ Papers across all procedures (*n* = 193) were obtained for full-text screening. Papers, eligible for inclusion, were categorised by the procedure. Ten papers that met the inclusion criteria related to oral surgery were retained for data extraction. The references of selected papers were screened and checked for any additional citations a priori and post hoc through which one additional paper was identified. Therefore, a total of 11 studies were included in the final review.

Key data items were extracted using a standardised data extraction form developed a priori and refined based on repeat pilot testing with a minimum of five publications and three data extractors. The data extracted for the overall study are presented in a table available at medRixiv,^23^ including study overview; dental procedures investigated; methodology; and relevant findings (related to the review outcomes). Detection methods for contamination were categorised as microbial, blood and other (non-microbial/non-blood). For studies where intervention was measured for its ability to alter aerosol spread, only data relating to the baseline or control (i.e. without the intervention effect) were extracted.

The quality assessment included a traffic light system developed to measure quality aspects for each study as well as the overall quality of across seven key domains measuring internal validity (bias, controls) and external validity (applicability, variability), as well as reporting standards. Each item on the tool was scored as red (standards not met), amber (standards partially met) or green (standards met) and any disagreement between reviewers was resolved through discussion with the wider team.^24^

## Results

### Included studies: overview

Eleven studies relating to oral surgery^34–44^ were included following the final review (Table 1). Most (*n* = 4) were conducted in Japan,^35,41–43^ followed by India (*n* = 3),^34,36,37^ with one each from the following countries: United Kingdom,^39^ Poland,^38^ Saudi Arabia^40^ and Spain.^44^ Publication dates ranged from 2008 to 2020, the research having been conducted up to 2019.

**Table 1:** Oral surgery: research methods.

Author, year (country)	AIMS	Methods						
		Setting 1. Study setting (single- and/or multi-chair) 2. Environmental factors (humidity, temperature, air conditioning)	Sample size (participant and/or procedure and/or sample details)	Procedure(s) 1. Cases 2. Control	Duration 1. Clinical procedures 2. Sampling	Equipment detail 1. Instruments 2. Irrigation 3. Mitigation (types of suction/evacuation) 4. PPE	Contamination 1. Type(s) 2. Site(s)	Sampling method
Ishihama et al., 2008 (Japan)	To evaluate the exposure of splattering contaminated with blood by the attending surgeon during outpatient surgery for an impacted mandibular third molar	1. Study setting: *Hospital* (not stated) 2. Environmental factors: Not stated	25 procedures (25 sets of PPE used by operators)	1. Cases: Surgical removal of the impacted tooth, alveoloplasty and transalveolar extraction (patients positioned at 45°, all treatments carried out by a single, right-handed surgeon) 2. Control: Control measures not used	1. Procedures: Recorded as <10, 10–20 or >20 min 2. Sampling: post procedure (duration not stated)	1. Instruments: Air motor handpiece (INTRAflex 2313 LN, KaVo, Germany) with steel round-bar at 12,000 r.p.m (standard); dental turbine handpiece (SUPERtorque LUX640, KaVo) with diamond point bar at 380,000 r.p.m. (standard); air motor handpiece with steel fissure bar 2. Irrigation: Water sprayed at 40–60 mL/min (standard) from the triple and single nozzles of the dental turbine handpiece and air motor handpiece 3. Mitigation: Suction at 80 L/min at 0.008 mpA standard setting by assistant 4. PPE: Surgical level PPE used	1. Type: Visible and imperceptible blood contamination on PPE 2. Sites: *Operators*: PPE used including operating gown and visor mask (areas covered: abdomen, femur, face shield, left arm, left forearm, mask, right forearm, right arm and thorax)	Visual check (with and without enhancers) of PPE used 1. Visible stains: visible check, count, location and size categorisation (small, 0.5 mm; large, 0.5 mm) 2. Imperceptible splatters: Leucomalachite green solution composed of 0.1 g of leucomalachite green (125660, Sigma-Aldrich, St. Louis, MO), 10 mL of acetic acid (017-00251, Wako, Japan), 0.5 mL of 30% hydrogen peroxide (081-04215, Wako) and 19.5 mL of distilled water (dilution experiment carried out to determine the sensitivity of the leucomalachite green solution to detect blood diluted up to 1:4000)
Ishihama et al., 2009 (Japan)	To assess the existence of floating blood-contaminated aerosols during outpatient surgery for a mandibular impacted third molar	1. Study setting: *Hospital* (Not stated) 2. Environmental factors: Not stated	132 procedures (100 procedures at 20 cm, 25 at 60 cm and 7 at 100 cm distances from the extraoral evacuator nozzle)	1. Cases: Impacted mandibular third molar surgery (patients positioned at 45°; operator not specified) 2. Control: Control measures not used	1. Procedures: 2–47.9 min of high-speed instrument use with a median time of 6.4 min in 79 cases 2. Sampling: post-procedure (duration not stated)	As per Ishihama et al., 2008 (see above) * An extraoral evacuator used at distances of 20, 60 and 100 cm	1. Type: Well-diluted and invisible blood stains 2. Sites: *Environment*: Aerosolised blood collected in the atmospheric samples collected by an extraoral high-volume evacuator system set at a distance of 20 cm for the first 100 cases, 60 cm for 25 cases and 100 cm for seven trial cases	Visual check (with enhancers) of filters placed on an air sampler Leucomalachite green solution composed of 0.1 g of leucomalachite green (125660, Sigma-Aldrich Inc., Missouri, MO, USA), 10 mL of acetic acid (017-00251, Wako Pure Chemical Industries Ltd, Osaka, Japan), 0.5 mL of 30% hydrogen peroxide (081-04215, Wako) and 19.5 mL of distilled water to test non-woven absorbable towel used as a filter on an extraoral high-volume evacuator system (3.0 m^3^/min at 5.0 kPa) (sensitivity of the filter towel determined as 96% of the American Society of Heating, Refrigerating and Air Conditioning Engineers (ASHRAE) test dust collection arrest under 430 Pa at initial pressure loss and 2.5 m/s of air velocity with a gravimetric method)
Wada et al., 2010 (Japan)	To evaluate the dissemination of blood and distribution of frequent contaminations, we investigated blood contamination on environmental surfaces of equipment in an outpatient procedure room	1. Study setting: *Hospital* (single-chair setting) 2. Environmental factors: Not stated	40 samples (sets of samples for light arm and bracket table arm from 20 cases)	1. Cases: Impacted mandibular third molar extraction (patients positioned at 45°; operator not specified) 2. Control: Control measures not used but surfaces disinfected with ethanol-based disinfectant cloths before each procedure	1. Procedures: Not stated 2. Sampling: post procedure (duration not stated)	As per Ishihama et al., 2008 (See above)	1. Type: Imperceptible blood contamination 2. Sites: *Clinical subsites*: Dental chair light arm and bracket table arm (described as low-touch areas)	Visual check (with enhancers) of absorbent wipes used for sampling Leucomalachite green solution composed of 0.1 g of leucomalachite green (125660, Sigmae Aldrich Inc., Missouri, MO, USA), 10 mL of acetic acid (017-00251, Wako Pure Chemical Industries Ltd, Osaka, Japan), 0.5 mL of 30% hydrogen peroxide (081-04215, Wako) and 19.5 mL of distilled water captured to test ethanol sterile absorbent cotton used to wipe down the environmental surfaces
Yamada et al., 2011 (Japan)	To clarify whether blood-contaminated aerosols were existent and floating in the air during dental procedures and to evaluate the effect of an extraoral evacuator system	1. Study setting: Hospital (Unclear but photograph included suggests multiple-chair setting) 2. Environmental factors: Not stated	226 procedures (52 impacted third molar extraction; 61 crown preparation; 47 inlay preparation; 66 scaling cases)	1. Cases *Oral surgery procedure*: Impacted third molar extraction Other treatment procedures: Full-crown preparation, black class II cavity preparation (in the proximal surfaces of molar and premolar) and scaling as cases that can induce bleeding. (patients positioned in a horizontal position, operator not specified) 2. Control: 19 inlay cavity preparation (black class I) conducted as bleeding is not induced	1. Procedures: Not stated 2. Sampling: During the procedure (duration not stated)	1. Instruments: High-speed rotating instrument and ultrasonic scaler 2. Irrigation: Not stated 3. Mitigation: Suction used but details unclear *An extraoral evacuator used for air sampling at distances of 50 and 100 cm PLUS a second extraoral evacuator at a distance of 100 cm 4. PPE: Unclear	1. Type: Blood-contaminated aerosol 2. Sites: *Environment*: 50 cm away from patient’s mouth and 100 cm away from patient’s mouth; and both 50 and 100 cm behind the patient’s mouth	Visual check (with enhancers) of filter on air sampler(s) Leucomalachite green solution (composition and dilution not stated) used to test filter placed on extraoral air evacuator (Free Arm FORTE-S, Tokyo Giken) 3.0 m^3^/min air at 5.0 kPa (sensitivity of the filter towel determined as 96% of the American Society of Heating, Refrigerating and Air Conditioning Engineers (ASHRAE) test dust collection arrest under 430 Pa at initial pressure loss and 2.5 m/s of air velocity with a gravimetric method)
Al-Eid et al., 2018 (Saudi Arabia)	To identify the extent of visually imperceptible blood contamination of the different surfaces of the oral surgery clinic and the PPE used therein, using forensic luminol	1. Study setting: *Hospital* (Unclear) 2. Environmental factors: Not stated	30 participants (details not provided)	1. Cases: Removal of one or both mandibular first molar (all treatments carried out by a single surgeon) 2. Control: Control measures not used but all clinical subsites disinfected before each procedure and tested	1. Procedures: 25–60 min (mean 40 min; SD 7.88; range 25–60 min) 20 procedures lasted >40 min out of 30 2. Sampling: Post-procedure (duration not stated)	1. Instruments: Rotary handpiece 2. Irrigation: Saline irrigation 3. Mitigation: Low-volume suction 4. PPE: Surgical level PPE used	1. Type: Imperceptible blood contamination 2. Sites: *Operator and assistants*: PPE included surgical gown, sterile gloves, face masks, eyewear, head cap and shoe cover *Patients*: PPE included head cap, eyewear and drape *Clinical subsites*: Tabletop for files and stationery; table for instruments and disposable; flooring behind the dental chair (including the operator’s and assistant’s chairs); instrument tray and handpiece unit; operating light and dental chair armrests; cuspidor and suction unit; and flooring in front of dental chair	Visual check (with enhancers) of PPE used and clinical subsites Luminol reagent spray (luminol blood detection reagent, TRITECH Forensics, Southport, North Carolina, USA) and isolation of the room from all light sources. Blacklight used to detect chemiluminescence to confirm the presence of traces of blood contamination (two calibrated investigators carried out a visual check for contamination)
Aguilar-Duran et al., 2020 (Spain)	Determining the prevalence of blood particles on masks with visors and surgical caps in oral surgery procedures and establishing the main risk factors for blood spatter	1. Study setting: *Hospital* (unclear) 2. Environmental factors: Not stated	216 samples (sets of caps and face masks used by surgeons and assistant for 108 procedures)	1. Cases: Extraction of impacted or erupted teeth, implant placement, extraction (non-surgical) (treatments carried out by multiple post-graduate trainees) 2. Control: Control measures not used	1. Procedures: Reported as ≥ or <30 min 2. Sampling: Post procedure (duration not stated)	1. Instruments: *Tooth extraction*: High-speed air-turbine handpiece or low-speed electric straight handpiece *Implant placement*: Electric contra-angled handpiece 2. Irrigation: *Tooth extraction*: Water cooling for high-speed air–water turbine and external cooling using a syringe for low-speed electric handpiece *Implant placement*: Saline cooling incorporated in the handpiece 3. Mitigation: Not stated 4. PPE: Surgical level PPE used	1. Type: Visible and invisible blood splatter 2. Sites: *Operators and dental assistants*: PPE used included facial masks with a visor (outer and inner sides) and surgical caps (outer side only)	Visual check (with and without enhancers) of PPE used 1. Visible blood contamination: Visual screening and count of blood splashes for each site 2. Imperceptible blood contamination: Kastle–Meyer reagent (two drops) plus two drops of 6% hydrogen peroxide added after 5 s (visual checks for contamination carried out by a single investigator)
Hallier et al., 2010 (UK)	To measure the levels of bioaerosol associated with dental procedures and to determine if these could be reduced in the local environment by use of the IQAir system both before and during certain types of dental procedure	1. Study setting: *Hospital* (single-chair setting for oral surgery and multiple-chair setting for other procedures) 2. Environmental factors: Clinic windows closed, no air conditioning systems, or fans on. Room temperature in all three clinical areas was between 21 and 24 °C	Eight participants [bioaerosol measured for each treatment (×4) for 2 cases (with without ACS) and plate change every 10 min. Between 5 and 9 bioaerosol samples collected. Fifteen separate bioaerosol samples at baseline] *Total number of samples = unclear	1. Cases: *Oral surgery procedure*: Extraction (non-surgical) *Other treatment procedures*: Cavity preparation, history and oral examination and ultrasonic scaling (all eight treatments carried out by eight different dental students) 2. Control: 15 baseline sampling performed during the weekend, with no dental treatment being undertaken	1. Procedures: Not stated 2. Sampling: During the procedure, plates replaced every 10 min	1. Instruments: *Scaling*: Ultrasonic Scaler (with high-volume aspiration) *Cavity preparation*: Air rotor high-speed dental handpiece *Extraction*: Not stated 2. Irrigation: *Scaling*: Not stated *Cavity preparation*: Not stated *Extraction*: Not stated 3. Mitigation: *Scaling*: High-volume aspiration *Cavity preparation*: Not stated *Extraction*: Not stated *Air cleaning system used for cases but not for controls PLUS an air sampling pump placed at a distance of 20 cm 4. PPE: Not stated but implied regular PPE used	1. Type: Bacterial 2. Sites: *Environment*: 20 cm away from the dental chair	Microbiological assessment of settle plates on an air sampler Sampling pump calibrated to 2.7 mm of water pressure at a flow rate of 100 L/min every 30 min for all procedures Blood agar plates incubated at 37 °C for 48 h under aerobic conditions (clinic windows closed, no air conditioning used during procedures, temperature maintained between 21 and 24 °C and air cleaning system used)
Jimson et al., 2015 (India)	To assess the bacterial composition of aerosols formed during surgical procedures	1. Study setting: *Hospital* (single-chair setting) 2. Environmental factors: Not stated	120 samples (4 samples for each of the 30 procedures)	1. Cases: Surgical removal of impacted mandibular third molar (treatments carried out by surgeon-unspecified) 2. Control: Two petri dishes exposed for 20 min before each procedure	1. Procedures: Not stated 2. Sampling: During the procedure for up to 20 min	1. Instruments: Surgical bur and handpiece 2. Irrigation: Not stated 3. Mitigation: Not stated 4. PPE: Not stated	1. Type: Bacterial 2. Sites: *Operators and assistants*: ‘Near surgeon,’ ‘near attendant’ *Patients*: Patient’s chest *Clinical subsites*: Instrument trolley	Microbiological assessment of settle plates Blood agar plates (20 min exposure during procedure) incubated at 37 °C for 24 h under aerobic conditions
Janani and Kumar et al., 2018 (India)	To determine the level and type of bacterial contamination presents on disposable surgical dental care clothing worn over scrubs of dental students to assess the risk of spread of nosocomial infection in a dental institution	Hospital (unclear) 2. Environmental factors: Not stated	135 samples (three swabs collected and cultured at the end of each of the 45 procedures) * Equivalent sites were swabbed at the beginning of each procedure but not reported	1. Cases: Surgical removal of impacted tooth, alveoloplasty, transalveolar extraction (treatments carried out by multiple post-graduate trainees) 2. Control: Samples collected at the beginning and at the end of each procedure	1. Procedures: Not stated 2. Sampling: Post-procedure (duration not stated)	1. Instruments: Not stated 2. Irrigation: Not stated 3. Mitigation: Not stated 4. PPE: Surgical level PPE used	1. Type: Bacterial 2. Sites: *Operators*: PPE used included surgical gown covering neck (collar), sleeve (cuff) and chest area	Microbiological assessment of swabs used for sampling Blood agar culture medium plate incubated at 37 °C under microaerophilic conditions (5% CO_2_) for 24 h
Kobza et al., 2018 (Poland)	To analyse the number of colony-forming units (CFUs) in bioaerosols and assess whether exposure limits are exceeded. Objective: To measure the concentration of bacteria and fungi in aerosols, in rooms where oral surgery was performed using high-speed instruments	1. Study setting: *General practice* (single- plus multiple-chair setting) 2. Environmental factors: Not stated	Not stated	1. Cases: Oral surgery procedure not specified (treatments carried out by dentists- unspecified) 2. Control: Air samples taken outside the dental practice before and during the working day	1. Procedures: Not stated 2. Sampling: During the procedure (duration not stated)	1. Instruments: High-speed ‘instrument' 2. Irrigation: Not stated 3. Mitigation: Not stated *An extraoral evacuator used at a distance of 30–60 cm for air sampling 4. PPE: Not stated	1. Type: Bacterial and fungal 2. Sites: *Environment*: 30–60 cm from surgical site	Microbiological assessment of filter on an air sampler Filter placed on an extraoral air evacuator used for morphological and microscopic analysis Bacteria: Morphological analysis using Tryptic Soy Agar base with cycloheximide added to inhibit fungal growth and microscopic analysis Fungi: Morphological analysis using Malt Extract Agar base and microscopic analysis
Divya et al., 2019 (India)	To evaluate the aerosol and splatter contamination from various minor oral surgical procedures and to assess the risk of spread of nosocomial infection in our dental institution	1. Study setting: *Hospital* (multiple-chair setting) 2. Environmental factors: Not stated	180 samples (six agar plates for each of the 30 patients; 10 alveoplasty, 10 transalveolar extraction, 10 surgical removals of impacted tooth)	1. Cases: Surgical removal of impacted tooth, alveoloplasty, transalveolar extraction (treatments carried out by operators- unspecified) 2. Control: Control measures not used	1. Procedure: Not stated 2. Sampling: During the procedure for 30 min	1. Instruments: High-speed handpiece 2. Irrigation: Water spray 3. Mitigation: High-volume evacuation used PLUS pre-procedural mouth rinse with chlorhexidine used before each procedure 4. PPE: Surgical level PPE used	1. Type: Bacterial 2. Sites: *Patients*: ‘On patient’ *Clinical subsites*: Instrument trolley and areas of the dental cubicle, including right middle cubicle, left middle cubicle, right corner and left corner	Microbiological assessment of settle plates Nutrient agar plates (30 min exposure during procedure) incubated at 37 °C for 24 h

The majority of identified studies (*n* = 9) were observational,^34–38,40,41,43,44^ in which the level of contamination and pattern of spread were two principal outcomes explored, while two studies were interventional, aimed at exploring various interventions to reduce the amount of aerosol (hence contamination) generated by oral surgery procedures.^39,42^ The latter also included additional dental procedures (periodontal and restorative).^39,42^ All the studies used clinical settings (ten hospitals and one general practice) to address their research question. The study by Kobza et al.^38^ carried out in general dental practice involved active air quality sampling in an oral surgery setting.

While all studies (*n* = 11) involved routine clinical care, the settings for oral surgery appeared mixed with three using single surgeries,^35,36,39^ one involving both single and multiple-chair facilities^38^ and two multiple,^34,42^ while the majority did not report this information.^37,39–41,43,44^ Only five studies^34,37,40,41,44^ formally reported staff wearing surgical level PPE involving gowns as well as masks/visors and gloves used by operator and/or assistants and in two the same processed were indicated.^35,43^

There was limited evidence on the environment in relation to air conditioning and humidity, with only the study by Hallier et al.^39^ providing details.

### What do the studies look at?

#### People

Five studies looked at the contamination of staff involved in the procedure,^36,37,40,41,44^ all of which considered the operators (surgeons and post-graduate trainees). Three studies also included assistants.^36,40,44^ Methods involved either swabbing,^37^ or examination of PPE,^40,41,44^ or the use of settle on plates near operators and assistants.^36^ Al-Eid et al.^40^ was the only study that considered contaminations across patient, operator, assistant and clinical subsites, using visual checks under chemiluminescence.

#### Clinical environment

Eight studies looked at the contamination of the wider clinical environment;^34–36,38–40,42,43^ two of which used settle plates,^34,36^ one assessed contamination using absorbent wipes.^35^

#### Air

Four studies examined air contamination.^38,39,42,43^ Hallier et al.^39^ actively examined air contamination at 20 cm; three further studies used extraoral evacuators fitted with filters to assess imperceptible blood contamination at distances ranging from 20 to 100 cm.^38,42,43^

#### Definitions

Divya et al.,^34^ Ishihama et al.^41^ and Jimson et al.^36^ defined ‘splatter’ as airborne particles >50 μm, which concurred with our definition. None of the studies provided a definition of aerosol, although Jimson et al.,^36^ recognised that aerosols <50 μm may be airborne for some time and that the nature and diameter of aerosol differ before, during and after a procedure. None measured the size of particles during their research.

#### Type of contamination

Finally, six studies examined blood contamination,^35,40–42,44,45^ visually and using standardised reagents,^46–48^ such as leucomalachite green, Kastle–Meyer and luminol. Four considered bacterial contamination culturing bacteria^34,36,37,39^ and one both bacterial and fungal,^38^ on a range of media.

#### Timing

In relation to timing, studies either collected splatter during or at the end of procedures and or actively sampled air for aerosols during procedures. Sampling of settled droplets or aerosol was conducted during (*n* = 5)^34,36,38,39,42^ or after (*n* = 6) the procedure.^35,37,40,41,43,44^ Sampling duration, where stated, ranged from 10 to 30 min during the surgical procedure. Sampling for visual and occult blood was conducted post surgery.

#### Controls

Baseline measurements and/or controls were used by some studies, although they differed from sampling at weekends when no dental treatment was being undertaken;^39^ to sampling before procedure^36^ (albeit not always reported in results);^37^ sampling outside the dental practice before and on the working day;^38^ and during a control procedure (class I cavity preparation), which did not involve the risk of blood contamination.^42^

#### Oral surgery procedures

Out of the 11 studies reviewed, nine considered contamination in relation to surgical removal of impacted teeth,^34–37,40–44^ generally third molars, including alveoplasty and transalveolar extraction, while one did not verify the ‘oral surgery’ procedure.^38^ One also included wider oral surgery procedures, including dental implant placement,^44^ while two others provided comparisons across dentistry with the inclusion of other dental procedures.^39,42^ Very small numbers of dental extractions (*n* = 9 in total) were considered within two papers by Aguilar-Duran et al.^44^ and Hallier et al.^39^

It was generally explicitly stated, and otherwise implied, that rotary instruments (handpieces) were involved in bone removal during the surgical procedure. Nine specified high-speed or slow/medium-speed surgical handpieces,^34–36,38,40–44^ five of which additionally reported the use of air-rotors to support tooth crown sectioning or preparation,^35,41,42,44,45^ six explicitly involved water coolant,^34,35,40,41,43,44^ and five reported the use of suction/evacuation.^35,40–43^ There was very little detailed information on water rate and suction/evacuation to facilitate comparison across studies.

#### Duration of procedures

Of the five studies that reported treatment length, the range was 2–60 min. Several considered the level of contamination in relation to the length of surgical treatment at 10-, 20-, 30- or 40-min thresholds.

### Quality assessment

The quality assessment for the studies showed a mixed picture for each of the seven domains; most studies scored “high” for two domains notably relevant to routine clinical dentistry (*n* = 11) and declaration of conflict of interest (*n* = 6); “moderate” for four domains including study funding (*n* = 6); sample size (*n* = 7), procedure description (*n* = 5) and outcome reporting (*n* = 5). The score for controls ranged from “high” (*n* = 2) to “low” (*n* = 2).

Sensitivity scoring for all studies looking at microbiological contamination (bacteria and fungi) were generally low (*n* = 5).^34,36–39^ Sensitivity scoring for studies looking at visible and imperceptible blood contamination generally ranged from moderate (*n* = 2)^35,43^ to high (*n* = 3),^40,41,44^ one study was low.^42^

### Outcomes

Outcomes will be examined in order of proximity to patient care, starting with the patient themselves and moving to the air of the dental operatory (Table 2). Given the heterogeneity of the data obtained, the results are presented in a narrative review and it is important to note that given the lack of universally agreed definitions, we report the terminology used in the original publications.

**Table 2:** Quality assessment including sensitivity.

### Overview of studies at people (patient, operator and assistant) and surface level

Overall, seven studies considered contamination at the patient, operator, assistant level and level of contamination of the surfaces in the dental operatory.^34–37,40,41,44^

#### Procedure overview

Ishihama et al.^41^ studied operator PPE on impacted mandibular third molar removal surgery (25 participants) on cases of mixed complexity, by one surgeon, using air motor handpiece for alveolar bone reduction and root sectioning and a dental turbine handpiece with a diamond point bar for sectioning the tooth crown. Coolant and/or water irrigation were used for all three procedures and aspiration was provided. Visible splatter and imperceptible blood contamination of the operator gown and visor mask covering areas, including abdomen, femur, face shield, left arm, left forearm, mask, right forearm, right arm and thorax, was measured using a standard reagent.

Jimson et al.^36^ examined aerosol for the presence of bacteria produced during surgical removal of impacted mandibular third molars involving a surgical bur and handpiece (30 patients). There was no mention of irrigation or the use or aspirators. Blood agar plates were used to measure the microbiological contamination on the instrument trolley (compared with near the surgeon and assistant and the patient’s chest). The blood agar plates were kept open for 20 min in total including the time of the surgical procedure and tested for aerobic bacteria.

Al-Eid et al.^40^ examined blood contamination of PPE, including sterile gloves, face masks, eyewear, surgical gown and head cover worn by the surgeon and assistant, during standard oral surgery procedures (no complications) conducted by the same surgeon (mean duration 40 min) involving rotary instruments (with irrigation) and low-vacuum suction (30 patients). Luminol reagent was sprayed directly onto PPE left behind and clinical subsites to check for non-visually detectable blood contamination (chemiluminescence was assessed by agreement of two trained examiners).

Janani and Kumar^37^ examined clothing of the operators; they conducted extraction and minor oral surgical procedures (45 participants), including impaction, transalveolar extractions and alveoloplasty. Sterile swabs were used to collect microbiological samples from the surgical clothing worn by post-graduate dental students carrying out the oral surgery procedure. Samples from areas including near the neck region, sleeve and chest area of the surgical clothing were collected and bacterial counts measured; however, the type of bacteria was not analysed.

Divya et al.^34^ investigated the contamination of aerosols and splatter on patients, instrument trolley and in standard locations (including right middle cubicle, one in the left middle cubicle and the right and left corners of the dental cubicle) during three different oral surgery procedures for 30 patients (10 alveoloplasty, 10 transalveolar extraction and 10 surgical removals of impacted tooth) involving high-speed handpieces (water spray) and high-volume evacuation. Nutrient agar plates were placed at five standard positions within the surgery (including on the patient) for 30 min during the procedure to measure the bacterial contamination; all patients had rinsed with chlorhexidine mouthwash prior to the procedure.

Wada et al.^35^ used disposable alcohol cotton to collect 40 samples from the dental chair light arm and bracket table (described as low-touch areas) and used leucomalachite green test to detect the percentage of positive reactions. They examined surface contamination of the dental operatory settings during 20 oral surgery cases involving the standard procedure outlined by Ishihama et al.,^41^ involving handpieces (air motor and turbine, irrigation and aspiration).

Aguilar-Duran et al.^44^ considered visual and invisible blood splatter on the masks and caps of the operator and assistants for a range of cases involving impacted or unerupted teeth. In addition, they reported separately on seven procedures that involved extraction only, without a surgical procedure. It is the only paper to have included extractions, without bone removal, but does not specify if a mucoperiosteal flap was raised.

### Outcome: patients

Three studies examined contamination of the PPE provided to protect patients, namely the chest drape, goggles and cap, ranging from low^34,36^ to intermediate^40^ sensitivity. Contamination from splatter and aerosol was measured using microbiological tests as well as standard chemical reagents, including visually imperceptible blood and aerobic bacteria.

#### Key findings relating to the outcome of interest

Divya et al.^34^ reported a higher bacterial count on the patient than the instrument trolley or at standard locations within the dental cubicle, showing decreasing bacterial counts with distance from the patient. There was evidence that the type of procedure influenced splatter, reporting that alveoloplasty resulted in greater levels of contamination than transalveolar extraction.

Al-Eid et al.^40^ reported visually imperceptible blood contamination of all patient chest drapes and 93.33% (28/30) of patient protective eyewear during standard minor oral surgery procedures. Procedures involved bone removal and sectioning of teeth by a rotary handpiece, with irrigation and low-volume suction and lasted an average of 40 min. The level of contamination was identified using a luminol reagent and was confirmed by two trained researchers. There was no evidence of blood contamination on the head caps of patients.

Jimson et al.^36^ detected bacterial growth on all 30 aerobically incubated plates positioned on the chest of the patient during surgical removal of impacted mandibular third molars involving a surgical bur and handpiece. There was no mention of irrigation. Blood agar plates on patient’s chest were kept open for 20 min in total including the time of the surgical procedure. Although there were differences between sites, the level of contamination as determined by the mean number of CFUs post surgery was not significantly different between the patient’s chest area (0.433; ±0.194) and other sites measured.

In summary, there was evidence of microbiological and blood contamination to the patients’ chest and face. The single study investigating the patients’ head (cap) did not identify blood contamination. All studies involving oral surgery procedures using drills of variable speeds, with/without reported irrigation, and including the use of suction (high/low/none stated) found contamination (visually imperceptible blood and bacteria) on the patient’s chest, and the one study that examined patient eye protection identified visually imperceptible blood in most cases.

### Outcome: operator/assistant

Three studies^40,41,44^ looked at visible and invisible blood contamination of the PPE during oral surgery procedures, while two^36,37^ examined microbiological contamination of PPE. Studies were of mixed quality: high,^41,44^ intermediate^40^ and low.^36,37^

#### Key findings relating to the outcome of interest

Ishihama et al.^41^ reported that the largest number of *visible stains* were the right forearm (*n* = 538), face shield (*n* = 326) and thorax (*n* = 127) region of the surgeon. Both the surgical gown and visor mask had evidence of higher *invisible* blood contamination (88% and 75%, respectively) compared with visible stains (64% and 60%, respectively). Surgical procedures lasting 20 min or more produced more evidence of blood contamination on surgeon’s PPE.

Aguilar-Duran et al.^44^ also found a positive correlation between the length of the procedure and the level of contamination with blood on face masks and caps of the operator and assistant when using Kastle–Meyer test. The majority of cases lasted 30 min or more (*n* = 158) and the level of blood contamination during these procedures was significantly higher compared with shorter procedures (*n* = 44) (50.6 ± 47.2–54.1%, cf. 29.6 ± 26.7–32.4%; *p* = 0.012). In terms of contamination, surgeons were found to have significantly more blood splatter than assistants (66.3 ± 63.3–69.4%, cf. 25.7 ± 23.1–28.4%; *p* = 0.001). Also, blood-contaminated splatter was significantly higher for the cases (*n* = 22) using high-speed air-turbine handpieces (77.3 ± 74.9–79.7%; *p* = 0.002) compared with those surgical cases (*n* = 136) using low-speed handpieces (45.6 ± 42.2–49.0%; *p* = 0.002). It was lowest for implant placement cases (*n* = 44) using contra-angle handpiece (31.8 ± 28.8–34.8%; *p* = 0.002). Surgeons were more likely to have splatter than assistants; also, 40% of the clinicians with positive blood contamination were unaware of the splatter. In terms of PPE contamination, the Kastle–Meyer test detected contamination on 33.2% (30.1–36.2) of the face masks, 37.6% (34.4–40.9) on the external part of the visor and 8.4% (7.4–9.5) on caps used. Interestingly, 4% (3.4–4.5) of the visors had blood contamination internally, none of which was detected by visual inspection alone. Seven extractions investigated by Aguilar-Duran et al.^44^ did not require any rotary instruments and were excluded from the above analysis; nonetheless, blood contamination was detected in 21% of the 14 PPE sets analysed (caps and facial masks for surgeons and assistants) (21.43 ± 19.1–23.8%).

Similarly, Janani and Kumar^37^ noted 100% bacterial contamination on the disposable surgical care clothing (gown) used by surgeons during minor oral surgery procedures. They also found that the bacterial counts were higher in cultures obtained from the sleeve cuffs of the surgical dental care clothing (37%), followed by the chest (34%) and the neck region (29%), with similar trends across each surgical procedure type. Overall bacterial colony counts were higher for alveoplasty (39%), compared with impaction (35%) and transalveolar extraction (26%).

Jimson et al.^36^ found bacterial growth on all 30 plates placed near the operator and the attendant for the presence of bacteria produced during surgical removal of impacted mandibular third molars. Although there were differences between sites, the level of contamination as determined by the mean number of CFUs post surgery was highest near the surgeon (0.468; ±0.218), followed by the area near dental attendant (0.448; ±0.236), and lowest on the trolley (0.383; ±0.168), with the level of contamination near the surgeon significantly higher than on the instrument trolley (*p* ≤ 0.001).

Al-Eid et al.^40^ detected bloodstains on all PPE worn by the clinical team (as well as patients), except the head caps and shoe covers. It was present on ALL the gloves and face masks of the operator and the vast majority of their protective eyewear (86.7%), gowns (73.33%) and handcuffs of aprons (46.7%). Blood contamination was also on ALL gloves (100%), most face masks (80%), protective eyewear (80%), surgical gowns (67%) and to a lesser extent in the handcuffs of the aprons (40%) worn by the assistant. A significant relationship was present between the length of the surgical procedure and frequency of blood contamination of the handcuffs of the aprons of both surgeon and assistants, at the 40-min threshold (*p* < 0.01).

In summary, the above studies highlighted a risk to surgeon operators, followed by assistants. Contamination of personal protective clothing (PPE) was generally present; it was highest on masks, visors and gowns, in particular, the gloves and cuffs of the surgical gown/apron.^37,40,41,44^ The evidence for contamination of surgical head cap was variable, with one study reporting low levels of contamination^44^ and another reporting no contamination of head caps and shoe covers used by surgeons and their assistants during oral surgery procedures.^40^ The one study with the most extensive analysis of PPE indicated contamination of the abdomen and femur.^41^

### Outcome: surface contamination

Four studies^34–36,40^ looked at microbiological contamination levels and spread of visible blood- and invisible blood-contaminated aerosols across various areas and surfaces, all within 1 m from the surgical site. Studies ranged from intermediate,^35,40^ to low,^34,36^ sensitivity.

#### Key findings relating to the outcome of interest

Divya et al.^34^ reported that bacterial counts on the instrument trolley were second only to the patient for all the minor oral surgical procedures. Within the dental cubicle, the left middle cubicle had a significantly higher count compared with the right middle cubicle (*p* < 0.05). High-volume evacuation was used but its location was not described. Bacterial counts decreased with distance from the patient. Of the surgical procedures examined (ten cases each: alveoplasty, transalveolar extraction and surgical removal of impacted tooth), alveoplasty resulted in greater levels of contamination than transalveolar extraction.

Jimson et al.^36^ detected bacterial growth from all 30 cases near the instrument trolley for the presence of bacteria produced during surgical removal of impacted mandibular third molars involving a surgical bur and handpiece. There was no mention of irrigation. Blood agar plates placed near the trolley were kept open for 20 min in total including the time of the surgical procedure. The nature of the bacteria appeared to differ by location, albeit that streptococci and staphylococci were most common in all locations. Although there were differences between sites, the level of contamination as determined by the mean number of CFUs post surgery was significantly lower near the instrument trolley (0.383; ±0.168), compared with near the surgeon (0.468; ±0.218) (*p* ≤ 0.001). There was a significant difference in the level of contamination of the room during surgery compared with beforehand as determined by the mean number of CFUs on the instrument trolley (*p* < 0.0001).

Similarly, Al-Eid et al.^40^ reported the presence of blood contamination (presumptive) across different clinical subsites including the instrument tray and handpiece unit (100% cases), operating light and dental chair armrests (100% cases), cuspidor and suction unit (100% cases) and the flooring below the patient’s headrest (86.67% cases).

Wada et al.^35^ reported that blood presumptive tests showed surface contamination even in minimal hand-contact areas such as the dental chair light arm and bracket table arm. Samples were collected from the light arm and bracket table arm across 20 clinical cases, one for each site (40 samples). Of the 20 samples from the light arm, 16 (80%) showed positive results for the blood presumptive test. In addition, of the 20 samples from the bracket table arm, 15 (75%) were positive. Three cases tested negative for blood overall (Table 3), accounting for six of the nine negative results.

**Table 3:** Oral surgery: outcomes measured and key findings.

Author, year (country)	AIMS	Outcomes measured					Key findings
		Operator	Assistants	Patients	Operatory	Environment	
Ishihama et al., 2008 (Japan)	To evaluate the exposure of splattering contaminated with blood by the attending surgeon during outpatient surgery for a impacted mandibular third molar	PPE for operator (surgeon): Localisation of stains on surgical gown and visor mask (areas included abdomen, femur, face shield, left arm, left forearm, mask, right forearm, right arm, thorax)	N/A	N/A	N/A	N/A	High incidence of blood contamination splatter to dental surgeon’s gown and visor/mask during oral surgery—both visible and invisible (imperceptible). Over 50% of stains were visible to the naked eye; on operating surgeon’s gown 24% visible and 76% not visible 469 visible bloodstain splatters on the gown and visor mask (296 small; 173 large), which came from 19 of 25 cases (76%). Volume varied from 0 to 78 small and 0 to 53 large splatters per operator Imperceptible blood splatters (*n* = 737) were present in 84% of gowns and 76% of visor masks. Presumptive tests to identify visible and invisible bloodstains (1206 reactions) were 2.57-fold greater than the visible stains (*n* = 469), and present in 88% of cases overall Imperceptible splatters covering areas, including abdomen, thorax, femur, left arm, left forearm, right forearm and right arm, as well as the face shield and masks worn by the surgeon. Largest number of stains present on right forearm = 538, face shield = 326 thorax = 127 regions of right-handed surgeons There was no significant difference on the presence or rate of occurrence in relation to the position and difficulty of the third molar; however, blood-contaminated splatter tended to increase as the procedure became more complicated and operation time increased
Ishihama et al., 2009 (Japan)	To assess the existence of floating blood-contaminated aerosols during outpatient surgery for a mandibular impacted third molar	N/A	N/A	N/A	N/A	Wider environment: Aerosolised blood (not splatter) in the atmospheric samples collected by the extraoral high-volume evacuator system placed at distances of 20 cm (*n* = 100 cases); 60 cm (*n* = 25 cases) and 100 cm (*n* = 7 cases), respectively, behind the patient	Blood-contaminated ‘mist’ was identified at 1 m distance from the oral surgical site, behind the patients who were seated at 450 The ratio of positive blood presumptive test for invisible mists: at a distance 20 cm behind patient mouth = 76% positive staining patterns varied from small dots to spread. A diffuse smudge pattern was observed in 23 cases (23%), and remaining 53 cases (53%) had individual positive dots that could be counted (range: 1–18) At distances of 60 and 100 cm contamination decreased to 60% (*N* = 15/25) and 57% (*N* = 4/7), which was not significantly different from the ratio at 20 cm (*χ*^2^ test: *p* = 0.1879) In the 60 cm distance trials, six cases (24%) showed a heavy positive reaction, while there were none categorised as heavy among the 100 cm trials. *χ*^2^ test showed no significant differences for occurrence ratio among the different third molar statuses At the distances of 60 and 100 cm, the proportion decreased to 60% (*N* = ¼, 15/25) and 57% (*N* = ¼, 4/7), respectively, although those were not significantly different from the ratio at 20 cm (*χ*^2^ test: *P* = ¼, 0.1879). In addition, the duration of high-speed instrument used had no significant effect on occurrence ratio nor on the degree of positivity (Kruskal–Wallis test, data not shown)
Wada et al., 2010 (Japan)	To evaluate the dissemination of blood and distribution of frequent contaminations, we investigated blood contamination on environmental surfaces of equipment in an outpatient procedure room	N/A	N/A	N/A	Clinical subsites: Dental chair light arm and bracket table arm (low-touch areas)	N/A	Forty samples from the light arm and bracket table arm were collected from 20 cases. Of the 20 samples from the light arm, 16 (80%) showed positive results for the blood presumptive test. In addition, of the 20 samples from the bracket table arm, 15 (75%) were positive. Only three cases displayed no positive reactions to the blood detection test on either surface
Yamada et al., 2011 (Japan)	To clarify whether blood-contaminated aerosols were existent and floating in the air during dental procedures and to evaluate the effect of an extraoral evacuator system	N/A	N/A	N/A	N/A	Wider environment: Aerosolised blood in the atmospheric air collected by a water-absorbent, non-woven towel set on the nozzle of two extraoral evacuator systems used at distances of 50 and 100 cm behind patients. In addition, the use of two air evacuators was tested at 50 and 100 cm behind the patient showed reduced contamination at 100 cm for surgical removal of third molars	Positive results on the presumptive test for blood were obtained in 92% of third molar surgery; 70% of crown preparation; 35% of inlay preparation; and 33% of scaling at a distance of 50 cm behind the mouth of the patient. Mean numbers of positive reaction dots on the test filter per time unit were 0.87/min for third molar surgery, 0.15/min for crown preparation, 0.14/min for inlay preparation and 0.17/min for scaling at a distance of 50 cm and differed significantly (*p* < 0.0001, one-way ANOVA); the number for third molar surgery was significantly larger than that for the other procedures (*p* < 0.0001 vs. crown preparation, inlay preparation and scaling, Scheffé post hoc test) At a distance of 100 cm, the mean number of positive dots on the test filter significantly decreased to 0.28/min for third molar surgery (*p* < 0.0001, Student’s *t* test). In other procedures, there was no significant difference of the mean numbers of positive dots per time unit between at distances 50 and 100 cm In relation to visible bleeding, dentists identified 46 cases with bleeding in crown and inlay preparation, and detected no bleeding for 62 cases. However, presumptive test revealed blood from 32% (20/62) of invisible bleeding cases
Al-Eid et al., 2018 (Saudi Arabia)	To identify the extent of visually imperceptible blood contamination of the different surfaces of the oral surgery clinic and the PPE used therein, using forensic luminol	PPE for the operator (surgeon): sterile gloves, face masks, eyewear, surgical gown, head cap and shoe cover	PPE for the assistant (dental assistant): sterile gloves, face masks, eyewear, surgical gown, head cap and shoe cover	PPE for patients: head cap, eyewear and chest drape	Clinical subsites: tabletop for files and stationery; table for instruments and disposable; flooring behind the dental chair (including the operator’s and assistant’s chairs); instrument tray and handpiece unit; operating light and dental chair armrests; cuspidor and suction unit; and flooring in front of dental chair	N/A	Clinical subsites: Blood contamination was detected in four subsites: 1. Flooring below the patient’s headrest: 26/30 cases (86.67%) 2. Instrument tray and handpiece unit: ALL cases (100%) 3. Operating light and dental chair armrests: ALL cases (100%) 4. Cuspidor and suction unit: ALL cases (100%) PPE: Blood contamination was detected in all the PPE except the head caps and shoe covers: 1. Oral surgeon: 100% contamination of the gloves + face masks. 8% Protective eyewear (*n* = 26/30), 73% surgical gowns (*n* = 22/30). 47% Handcuffs of the aprons (14/30) 2. DA: 100% gloves; 80% face masks and protective eyewear (*n* = 24/30); 67% surgical gowns (*n* = 20/30); 40% handcuffs of the aprons (*n* = 12/30) 3. Patient: 100% contamination of chest drapes 93% of the protective eyewear (*n* = 28/30) •A statistically significant interaction between surgical procedure time and the frequency of blood contamination in the handcuffs of the aprons of the oral surgeon and the DA (*p* < 0.01) was noted
Aguilar-Duran et al., 2020 (Spain)	Determining the prevalence of blood particles on masks with visors and surgical caps in oral surgery procedures and establishing the main risk factors for blood spatter	PPE for the operator (post-graduate trainees): Outer side of caps and inner and outside of facial masks used	PPE for the assistant (dental assistants): Outer side of caps and inner and outside of facial masks used	N/A	N/A	N/A	Visual check: Visual inspection revealed greater blood spatters on the external part of the visors, followed by the masks and minimal splashes on the caps. Presumptive tests for invisible blood stains: The Kastle–Meyer test detected blood in 28% of the samples (95% confidence interval [CI], 25.1–30.6%) that were classified as negative via visual inspection. In eight samples (3.96%), the test detected blood in the internal part of the visor, four of them linked to the use of a high-speed air-turbine handpiece (three samples from surgeons and one sample from an assistant) and the other four linked to the use of a low-speed electric straight handpiece (all of them from surgeons) Blood splashes were found more often from surgeons, although assistants also had positive samples. The use of a high-speed air-turbine handpiece produced the highest percentage of blood splash (77.3%), followed by a low-speed electric straight handpiece (45.6%) and a contra-angle handpiece 20:1 for implant placement (31.8%) Procedures beyond 30 min were more prone to have blood contamination. Forty percent of the clinicians were unaware of blood spatters
Hallier et al., 2010 (UK)	To measure the levels of bioaerosol associated with dental procedures and to determine if these could be reduced in the local environment by use of the IQAir system both before and during certain types of dental procedure	N/A	N/A	N/A	N/A	Wider environment: Air sampled at a distance of 20 cm from the dental chair using IQAir system at baseline and during procedures, with and without an air cleaning system	Bioaerosol levels increased during tooth extraction from a baseline of 9.1–66.1 CFU/m^3^ in the absence of any air cleaning system or air movement (air conditioning or open window). Activation of the air cleaning system (ACS) when the single surgery clinic was empty and no dental procedure being performed (control- weekend), produced a significant reducing in bioaerosol level from 9.1 to 2.5 CFU/m^3^ (*p* = 0.01). Use of an ACS during dental extraction resulted in a lower count during dental extraction: 37.0, cf. 66.1 CFU/m^3^ (*p* < 0.05) based on one clinical case Assessment of bioaerosol levels during the four dental procedures with the ACS in operation revealed a significant reduction in bacterial levels for all four procedures: statistically significant for cavity preparation (*p* = 0.018), ultrasonic scaling (*p* = 0.027) and tooth extraction (*p* = 0.036) The predominant microorganisms isolated during this study were *Staphylococcus* and *Micrococcus* species
Jimson et al., 2015 (India)	To assess the bacterial composition of aerosols formed during surgical procedures	Operator: ‘Near surgeon’	Assistant: ‘Near assistant'	Patients: ‘Patient’s chest'	Clinical subsites: ‘ Near instrument trolley'	N/A	There was a significant difference in the level of contamination before/after surgery for the wider controls test plates in the surgery. Although there were differences between sites, the level of contamination as determined by the mean number of CFUs post surgery (CFU/cm^2^) was highest near the surgeon (0.468; ±0.218), followed by the area near dental attendant (0.448; ±0.236) and lowest on the instrument trolley (0.383; ±0.168), with the level of contamination near the surgeon significantly higher than on the instrument trolley (*p* ≤ 0.008). Alpha-haemolytic streptococci are the predominant bacterium seen in all the 30 surgeries, followed by other bacteria. The nature of the bacteria grown appeared to differ by location, albeit that streptococci and staphylococci were most common in all locations. Bacteria grown on the blood agar plate near the surgeon and the patient were similar
Janani and Kumar, 2018 (India)	To determine the level and type of bacterial contamination present on disposable surgical dental care clothing worn over scrubs of dental students to assess the risk of spread of nosocomial infection in a dental institution	PPE for the operator (post-graduate trainees): Neck region (collar), sleeve and chest area of the surgical clothing	N/A	N/A	N/A	N/A	*Only post-surgery procedures can be reported as baseline count was not provided Bacterial colony counts were greater in cultures obtained from the sleeve cuffs of the surgical dental care clothing compared with the neck region (collar region) Bacterial colony counts cultured following alveoloplasty procedure were greater in number when compared to transalveolar extraction procedure Multi-chair setting: Bacteria CFU/m^3^ concentration during the procedure = 30 (360–500); fungi = 300 (0–330) Single-chair setting: Bacteria CFU/m^3^ concentration during the procedure = 490 (200–1190); fungi = 110 (40–220). The largest proportion of organisms in both of the dental surgeries were Gram-positive cocci, which ranged from 74 to 100% of the sample. The remainders were Gram-positive, rod-shaped bacteria and those creating endospores as well as non-porous bacteria The dominant fungi were Cladosporium and Penicillium types. The concentration of total bacterial and fungal aerosols was similar in both dental offices, and a significant increase was observed during dental treatment
Kobza et al., 2018 (Poland)	To analyse the number of colony-forming units (CFUs) in bioaerosols and assess whether exposure limits are exceeded Objective: To measure the concentration of bacteria and fungi in aerosols, in rooms where oral surgery was performed using high-speed instruments	N/A	N/A	N/A	N/A	Wider environment: 30–60 cm from the surgical site	Multi-chair setting: Bacteria CFU/m^3^ concentration during the procedure = 30 (360–500); fungi = 300 (0–330) Single-chair setting: Bacteria CFU/m^3^ concentration during the procedure = 490 (200–1190); fungi = 110 (40–220). The largest proportion of organisms in both of the dental surgeries were Gram-positive cocci, which ranged from 74 to 100% of the sample. The remainder were Gram-positive, rod-shaped bacteria and those creating endospores as well as non-porous bacteria. The dominant fungi were Cladosporium and Penicillium types
Divya et al., 2019 (India)	To evaluate the aerosol and splatter contamination from various minor oral surgical procedures and to assess the risk of spread of nosocomial infection in our dental institution	N/A	N/A	Patient: Contamination on patient	Clinical subsites: Contamination of instrument trolley	Wider environment: Contamination of right middle cubicle, one in the left middle cubicle and the right and left corners of the dental cubicle	The bacterial CFUs were higher on the patient’s chest and the instruments trolley used for all the minor oral surgical procedures. Bacterial colony counts were greater in cultures obtained from the left middle cubicle compared with the right middle cubicle and the results were statistically significant (*p* < 0.05). Bacterial colony counts cultured from dental cubicle following alveoloplasty procedure were greater in number when compared to transalveolar extraction procedure. Bacterial colony counts were greater in cultures obtained from the left middle cubicle compared with the right middle cubicle and the results were statistically significant (*p* < 0.05). Bacterial colony counts cultured from dental cubicle following alveoloplasty procedure were greater in number when compared to transalveolar extraction procedure

Overall, the studies reported the presence of blood and microbial contamination in surfaces surrounding the dental operatory following surgical procedures. They highlight the contamination to the dental operatory and clinical environment up to a distance of 1 m from the surgical site, with an inverse relationship of contamination to distance; however, none of the studies looked beyond this threshold. The instrument trolley was most frequently contaminated with bacterial and fungal species, as well as imperceptible blood. Even low-touch areas, as well as wider areas within the operatory room, such as the flooring under the patient’s head, were found to be commonly contaminated during oral surgery procedures.

### Overview of studies measuring air contamination

Four studies^38,39,42,43^ measured air contamination during oral surgery procedures, through active air sampling either by means of an extraoral evacuator placed at distances ranging from 20 to 100 cm with a nozzle filter, checking for presumptive blood contamination using standard reagents^42,43^ or microbiological detection with agar plates.^38,39^ Studies were of low,^38,39,42^ to moderate,^43^ sensitivity.

#### Procedure overview

Ishihama et al.^43^ used a single extraoral evacuator system at three distances (20 cm for first 100 cases, 60 cm for 25 cases and 100 cm for 7 trial cases) behind the patient to detect the presence of aerosolised blood during the removal of impacted mandibular third molars (100 participants) using standard procedures (motorised handpiece for bone removal; air turbine for tooth division; irrigation and aspiration) outlined in his earlier paper.^41^ Presumptive blood contamination (number of positive dots) was tested using leucomalachite green and hydrogen peroxide.

Kobza et al.^38^ carried out morphological and microscopic analysis of bacteria and fungi present in aerosol (undefined) generated during oral surgery procedures using high-speed instruments (details not provided). A single extraoral evacuator system was placed 30–60 cm from the surgical site to collect bioaerosol in what was considered the breathing zones of patients (in a single and multiple-chair surgery) and used Tryptic Soy Agar with cycloheximide, Malt Extract Agar, for microbiological analysis of the bioaerosol collected compared with controls (outside the practice and prior to patient care)

Hallier et al.^39^ conducted microbiological analysis (bacterial CFU count) in air sampled at 20 cm distance from the dental chair in single surgery to compare dental extraction, with and without an air cleaning system during a range of dental procedures. This was compared with sampling at the same clinic at a weekend when no procedures were being undertaken.

Yamada et al.^42^ used a presumptive test for blood at positions of 50 cm and 100 cm *behind* the surgical field and where aerosol ‘particle mist’ and splatter from an air turbine were ‘recorded’ as going upwards. Positive presumptive blood exposures were calculated for a range of procedures including surgical third molar extraction using high-speed rotating instrument. In an additional experiment, placement of a second evacuator at a distance of 100 cm, was used to examine the effect of two evacuators at differing proximity.

#### Key findings relating to the outcome of interest

Ishihama et al.^43^ found that presumptive blood contamination (number of positive dots) during oral surgery procedures was highest at the proximal location of 20 cm (76%), decreasing with distance (60% at 60 cm and 57% at 100 cm).

Yamada et al.^42^ found that the presumptive test for blood was 92% (12/13) for third molar removal at 50 cm behind the surgical field and 90% (35/39) at 100 cm from a zone where aerosol ‘particle mist’ and splatter from an air turbine was ‘recorded’ as going upwards. At a 50 cm behind the patient, the mean number of positive presumptive blood exposures (dots per unit time was highest for surgical third molar extraction (0.87/min) in comparison with other dental procedures such as crown preparation (0.15/min), inlay preparation (0.14/min) and scaling (0.17/min). In an additional experiment, placement of a second evacuator at a distance of 100 cm, the level of presumptive blood contamination (mean number of positive dots per unit time) decreased for third molar surgery (0.28/min) (*p* < 0.0001), but not other restorative and periodontal procedures. The level of presumptive blood contamination was significantly higher for third molar surgery when compared with other procedures (*p* < 0.0001).

Kobza et al.^38^ reported that the average level of microbiological contamination (bacteria and fungi) increased during patient care involving oral surgery procedures, compared with controls, in both single- and multi-chair settings. The dominant bacteria were Gram-positive cocci and rods and fungi were environmental fungi (*Cladosporium* and *Penicillium* species).

Hallier et al.^39^ in this study involving just two episodes of tooth extraction resulted in increased air contamination. Comparison between procedures was complicated by the fact that the other procedures were undertaken in multi-surgery facilities. Contamination increased during the surgical procedure and was significantly lowered by the use of an air cleaning system.

In summary, all four studies actively assessing aerosol reported air contamination (blood or microbiological) during oral surgery procedures at distances up to 1 m. it is important to note again that no study measured beyond 1 m and the limited research at this point did show contamination at the patient level, albeit decreasing. The research appears to have been focused around, or just above, the patient level. Overall, oral surgery-related procedures were associated with positive presumptive identification of blood aerosols, bacteria and fungi at distances up to 1 m, providing evidence that contamination levels increase as a result of surgery and decrease with distance from the surgical field and additional air evacuation. Within the findings, there is evidence of increased contamination with simple extractions, albeit very limited and/or poor/medium quality.

### Studies comparing contamination generated by oral surgery and other procedures (within the study)

Two studies provided comparisons across dentistry, with the inclusion of other dental procedures.^39,42^ Hallier et al.^39^ in a small study of low sensitivity suggested that extraction in single surgery setting may result in less aerosol than procedures involving cavity preparation, but similar to history and examination and ultrasonic scaling in a multi-surgery setting, with contamination levels being significantly lower at weekends and significantly reduced, but not eliminated, with the use of an air cleaning system.

Blood splatter with dental implant surgery was less than oral surgery involving surgical removal of teeth with a surgical handpiece.^44^ This was attributed to the higher working speed of the surgical handpiece and its external irrigation.

### Summary of the findings on aerosol contamination/spread associated with oral surgery procedures

In summary, the research in relation to oral surgery involving removal of teeth, generally third molars, found the risk of contamination to the patient (chest and face), operator (face, chest, arm/glove/cuff/abdomen/femur) and assistant, as well as the dental operatory and air environment. PPE used by surgeons and assistants (where stated) was mostly surgical, including gowns, and the research was conducted in a range of settings, mainly dental hospital outpatient facilities. Most evidence was of blood splatter (visible and imperceptible), while microbiological examination was limited to bacterial culture with aerobic incubation. Imperceptible blood splatter was significantly higher than visible stains. Very limited research evidence on extractions suggests that they may be of lower risk, but not without risk of contamination. Risks varied by type of procedure and instruments used, which increased with time and decreased with distance. Risks were reduced but not eliminated by external evacuators, where used.

## Discussion

All 11 studies included in this review found the risk of blood and microbiological contamination to patients, dental team and dental operatory present at some stage across all settings, procedures and distances during oral surgery procedures, including non-surgical tooth extraction. Considerable heterogeneity with regards to definitions, methodologies and outcomes was noted, with only two of the studies considering dimensions and definition of the particles measured. While contamination during oral surgery procedures showed significant risk during and as a result of procedures (verified by both microbiological analysis and standard chemical reagents); our review of their sensitivity suggests that the reported data represent a significant underestimation of the true levels of contamination because of their low sensitivity. Furthermore, given the limited methods of cultivation, the nature of the bacteria present has not been fully described, albeit that oral commensals were reported. Furthermore, none of the studies included the detection of viruses, which are now of major relevance. However, as bleeding is expected during oral surgery procedures, detection of blood in oral secretions reveals evidence of contamination, which will undoubtedly include SARS-CoV-2 in infected individuals.

In relation to blood contamination, the use of standard reagents for the presumptive identification of blood revealed more extensive contamination (aerosol) than indicated by visible blood (splatter), particularly where all disposable PPE were examined. These studies were of higher quality, and the assessment method was of medium to high sensitivity, suggesting that their findings are more robust. However, it is important to note that they found contamination in almost all sites measured (shoe covers being an exception in one study). In addition, there was blood contamination on the inner surfaces of masks used, which suggests the presence of blood aerosol.

There was evidence of aerosol in all locations examined. Yamada et al.^42^ sampled the air behind the patient and surgical site and found aerosolised blood. A further paper by Ishihama, not included in the review because it was conducted in an operating theatre rather than a dental operatory, found evidence of blood contamination at 3.8–4.6 m in the air conditioning unit, as a result of what was termed *floating blood mist*.^45^ Support for our findings is present in other reviews relating to AGPs in healthcare,^49^ and head and neck surgery.^50^ Furthermore, evidence is emerging that aerosol plumed from coughing and sneezing may be extensive, extending up to 6 m by sneezing,^16^ both of which can occur in the dental surgery.

In relation to procedures, there appeared to be a lower level of contamination from surgery involving implants compared with removal of third molars,^44^ and differences by technique for surgical removal of teeth, but none were contamination-free. While there is low-quality evidence on extractions, given the very low numbers involved, there was clearly some risk of aerosol.

Our findings strongly confirm the importance of wearing surgical level PPE to protect staff and patients from droplets and splatter during oral surgery procedures.^51^ However, since there is increasing evidence that SARS-CoV-2 may be transmitted via the airborne route, particularly over ‘intermediate’ distances,^17,52^ additional PPE is therefore required. Furthermore, given the risk of nosocomial infection,^8^ strict precautions in using PPE are important.

This research is an important reminder that patients are clearly at risk of contamination, and their protection from splatter, droplets and aerosol is important. Of course, much of the splatter during a procedure will include patient’s own bacteria, blood and viruses. Anything that is aerosolised has the potential to be inhaled by others and transmit infection between patients and to staff during the working day. This clearly requires further investigation, particularly given the evidence from Van Doremalen et al.^25^ that the virus can remain infectious for at least 3 h after aerosolisation. Furthermore, patients leaving the surgery having acquired splatter or droplets will carry and may shed them later, potentially putting others at risk of infection.

Significant limitations include the paucity of research in this field and the paucity of research with high sensitivity conducted to examine viruses and looking to settle over time. Furthermore, much of the research to date has involved a horizontal dimension, testing at the level of the operator and patient; however, it is clearly important to understand the vertical component of aerosols and their behaviour in relation to specific equipment and procedures, including coughing and sneezing.^39^ Also, the emerging evidence on the importance of the temperature, humidity ventilation and airflow within the environment^14^ requires further consideration in dental surgeries. Given that much oral surgery is conducted in primary dental care settings, particularly in countries such as the four nations of the United Kingdom, future research should also be conducted in a range of settings.

Despite the limitations, the available research evidence provides useful directions for future studies in the field and influences action strategies to ensure that we can establish a robust high-quality research evidence in support of infection control and risk mitigation in the oral sugery. A number of factors should be considered in the design and reporting of future studies to improve their sensitivity and reliability for informing future procedures and practice.

First, the environment of the operating room is an important factor,^53^ particularly the type and operating parameters of any mechanical ventilation. Introduction of fresh air or recirculation of air should be recorded along with the presence of any filtration or disinfection system. The number of air changes per hour should be measured. Humidity can have a significant impact on the survival of microorganisms in air and relative humidity should be recorded before and after the performance of the surgical procedure.

Second, the equipment used for the procedures themselves should be carefully described, particularly settings that affect aerosol production, such as the mixing of liquids with air. The type of suction devices should be recorded along with the volume of air extracted during use.

Third, regarding air sampling, the size of particles measured is critical in predicting the duration for which droplets and aerosol particles will remain airborne. The assessment of surface contamination is commonly performed using microbiological methods. Where bacteria are detected, the most sensitive methods should be used, as already discussed in this review. These include the use of blood-containing complex culture media incubated in an anaerobic atmosphere for at least 7 days. Direct molecular methods of bacterial detection and identification are now available based on probing for or sequencing bacterial DNA. Such methods are highly sensitive but will require any free DNA from dead bacteria present in the samples to be denatured before analysis.

Fourth, controls should helpfully include air sampling during long periods of non-working such as weekends,^39^ and investigate air contamination across the working day, as well as over the working week.

The impetus for this, and many other reviews, has clearly been the COVID-19 pandemic. It is critical that future research on infection control includes the study of viruses. This could include spiking body fluids with known amounts of non-pathogenic viruses. Detection of viruses is most easily accomplished by detection of their nucleic acid—DNA or RNA. Although useful, nucleic acid detection does not equate to infectivity. To assess the viability of viral particles over time, a viral culture will be required, which is a specialist area and will entail collaboration with virology specialists.

Chemical detection of dyes and fluorescent compounds or blood components themselves has been commonly used as a surrogate for contamination of air and/or surfaces with body fluids. Much of this research has been informed by forensic pathology.^46–48^ A key issue to consider is dilution and the potential for under- or over-representation of blood products.^47,48^

Finally, a power calculation should be performed to ensure that sufficient observations are made to yield statistically significant differences to be detected and clinically significant conclusions drawn.

## Conclusion

The studies included in this review, although generally of only low to medium quality, have highlighted the risk of contamination (microbiological, visible and imperceptible blood) to patients, dental team members and the clinical environment during oral surgery procedures, most notably removal of impacted third molars but also routine extractions. However, the extent of contamination has not been explored fully in three dimensions and over time. Variability across studies with regards to the methodologies used and outcome measures makes it difficult to draw robust conclusions. Nonetheless, it is clear that patients, operators and assistants are frequently contaminated as a result of oral surgery procedures. The wearing of full PPE is therefore appropriate, particularly given the potential risk of infection with respiratory viruses. The importance of appropriate doffing of contaminated clothing must be emphasised. Steps should be taken to minimise risk by good operator technique, supported by high-volume suction. There is a need for greater information on dental extractions in particular as oral surgery is indispensable; thus, having a good understanding of the nature and extent of contamination is vitally important. Further studies with improved methodologies and higher test sensitivity are required to validate these findings, along with greater consideration of pathogenic viruses.
